# Alteration of metabolite profiling by cold atmospheric plasma treatment in human myeloma cells

**DOI:** 10.1186/s12935-018-0541-z

**Published:** 2018-03-20

**Authors:** Dehui Xu, Yujing Xu, Ning Ning, Qingjie Cui, Zhijie Liu, Xiaohua Wang, Dingxin Liu, Hailan Chen, Michael G. Kong

**Affiliations:** 1 State Key Laboratory of Electrical Insulation and Power Equipment, Centre for Plasma Biomedicine, Xi’an Jiaotong University, Xi’an, 710049 Shaanxi People’s Republic of China; 2 The School of Life Science and Technology, Xi’an Jiaotong University, Xi’an, 710049 Shaanxi People’s Republic of China; 3 Frank Reidy Center for Bioelectrics, Old Dominion University, Norfolk, VA 23508 USA; 4 Department of Electrical and Computer Engineering, Old Dominion University, Norfolk, VA 23529 USA

**Keywords:** Cold atmospheric plasma, Multiple myeloma, Metabolite profiling, Mass spectrometry, KEGG analysis, Beta-Alanine metabolism

## Abstract

**Background:**

Despite new progress of chemotherapy in multiple myeloma (MM) clinical treatment, MM is still a refractory disease and new technology is needed to improve the outcomes and prolong the survival. Cold atmospheric plasma is a rapidly developed technology in recent years, which has been widely applied in biomedicine. Although plasma could efficiently inactivate various tumor cells, the effects of plasma on tumor cell metabolism have not been studied yet.

**Methods:**

In this study, we investigated the metabolite profiling of He plasma treatment on myeloma tumor cells by gas-chromatography time-of-flight (GC-TOF) mass-spectrometry. Meanwhile, by bioinformatic analysis such as GO and KEGG analysis we try to figure out the metabolism pathway that was significantly affected by gas plasma treatment.

**Results:**

By GC-TOF mass-spectrometry, 573 signals were detected and evaluated using PCA and OPLS-DA. By KEGG analysis we listed all the differential metabolites and further classified into different metabolic pathways. The results showed that beta-alanine metabolism pathway was the most significant change after He gas plasma treatment in myeloma cells. Besides, propanoate metabolism and linoleic acid metabolism should also be concerned during gas plasma treatment of cancer cells.

**Conclusions:**

Cold atmospheric plasma treatment could significantly alter the metabolite profiling of myeloma tumor cells, among which, the beta-alanine metabolism pathway is the most susceptible to He gas plasma treatment.

**Electronic supplementary material:**

The online version of this article (10.1186/s12935-018-0541-z) contains supplementary material, which is available to authorized users.

## Background

Multiple myeloma (MM) is a malignant tumor caused by abnormal proliferation of monoclonal plasma cells, accounting for 1% of all tumors and 13% of hematological malignancies [1, 2]. After the initial onset of remission, relapse will occur and only 25% of patients have a survival of more than 5 years after receiving chemotherapy [3]. It is characterized by an increase in abnormal plasma cells that produce monoclonal immunoglobulin and malignant proliferation in the bone marrow, causing fractures and bone marrow failure [4, 5]. The current clinical treatment of MM includes radiation therapy, bone marrow transplantation and chemotherapeutics treatment [6, 7]. Radiation therapy, however, will inevitably damage human normal cells while killing cancer cells. Bone marrow transplantation may result in postoperative autologous rejection. Chemotherapeutics may have serious side effects and usually lead to drug resistance. Therefore, MM is a refractory disease and new technology and treatment tools need to be developed for MM therapy.

Cold atmospheric plasma (CAP) is a new technology rapidly developed in recent years. It is produced under atmospheric pressure with low gas temperature and high activity of reactive species, which has aroused widespread concern especially in biomedical application, such as disinfection of bacteria, application in dermatology and dentistry, cell transfection, wound healing and cancer treatment [8–17]. It is widely reported that plasma could efficiently inactive tumor cells in various types of cancer, including lung cancer, leukemia, intestinal cancer, melanoma, cervical cancer, glioma, multiple myeloma, pancreatic cancer et al. [18–28]. The induction of apoptosis in cancer cells has been widely reported, and the mechanism of plasma-induced apoptosis is being increasingly understood. However, the effects of plasma on tumor cell metabolism have not been reported yet. Cell metabolism is a general term for a series of ordered chemical reactions that take place in the cells to survive. These reaction processes allow cells to grow and reproduce, maintain their functions and respond to the external environment, including the metabolism of matter and energy. Tumor cells provide a source of their aberrant proliferation through a systematic reprogramming of cellular metabolism [29, 30]. These changes in metabolism involve the production of energy required for cell division, the regulation of intracellular redox status, and the breakdown and synthesis of nutrients after ingestion, thereby altering the flux of metabolites inside and outside cells and redistributing them to the corresponding metabolic pathways, to meet the needs of maintaining the malignant transformation phenotype of cells [31]. Therefore, understanding the effects of gas plasma on tumor cell metabolism is of great significance. In this study, we explored the influence of gas plasma on tumor cell metabolism profiling for the first time. By metabonomics, we found that the metabolism of myeloma tumor cells was greatly changed after He plasma treatment. Notably, beta-alanine metabolism pathway was found to be the major target that was affected by gas plasma treatment, indicating that beta-alanine might play an important role in the interaction of gas plasma with tumor cells.

## Methods

### Gas plasma generation

In this study, we used a plasma jet which was described in our previous research to generate the cold atmospheric plasma. Characters of the plasma generation and electronic parameters were illustrated in our previous works [17, 28, 32]. The He plasma was generated at 10 kHz/8 kV with a He gas flow of 2 SLM.

### Cell culture condition

The LP-1 multiple myeloma cell line was used in this study. LP-1 cells were grown in Roswell Park Memorial Institute (RPMI) 1640 medium supplemented with 10% fetal calf serum, 100 U/mL penicillin, and 50 µg/mL streptomycin (Gibco-Invitrogen, Carlsbad, CA, 15140-122). The cells were cultured at 37 °C in an incubator (Thermo Scientific, Waltham, MA, USA) containing 5% CO_2_. The medium was refreshed 24 h before performing experiments.

### Cell viability assessment

Cell viability was measured by A Cell-Titer-Glo^®^ luminescent cell viability assay kit (Promega, Madison, WI, USA) which based on the production of ATP in viable cells. 100 μL of samples and 100 μL of Cell-Titer-Glo^®^ reagent were added to the opaque-walled plate and was incubated at room temperature for 10 min. The luminescence was detected by a microplate reader (Thermo Scientific Varioskan Flash, Waltham, MA, USA) with the protocol of “luminometric” measurement.

### Solvents and reagents

We bought L-2-chlorophenylalanine from Hengbai Biotechnology Co Ltd (Shanghai, China), while Methoxy amination hydrochloride (chromatographic grade), pyridine and chloroform (HPLC grade) were from Admas (Shanghai, China). Moreover, BSTFA (including 1% TMCS, v/v) was purchased from REGIS Technologies Inc (Morton Grove, IL, USA) and methyl alcohol (HPLC grade) was purchased from ANPEL Laboratory Technologies Inc (Shanghai, China). Saturated fatty acid methyl fat (C8, C9, C10, C12, C14, C16, C18, C20, C22, C24) was bought from Dr. Ehrenstorfer (Augsburg, Germany). Deionized water was used throughout this experiment (Thermo; Waltham, MA, USA).

### Sample collection

3 × 10^5^ cells/well in 300 μL of medium were seeded in 24-well plate, then the wells which treated with 40 s of He plasma were considered as plasma treatment group and the remains were control group, and each group had 5 duplicates/samples. After 24 h incubation, cells were collected and counted to ensure the cell number was about 1 × 10^7^ cells/sample. Cells were harvested by centrifugation at 4 °C for 5 min with the speed of 1200 rpm and washed with PBS three times at 4 °C for 3 min with the speed of 900 rpm. Then the cell pellet in EP tube was rapidly put in liquid nitrogen for 5 min and stored in − 80 °C refrigerator until analysis.

### Sample preparation

Before metabolite analysis, sample was mixed with 0.6 mL of extraction liquid (V_methanol_:V_Chlorofrom_ = 3:1) in 2 mL EP tube and 10 μL of L-2-chlorophenylalanine (1 mg/mL stock in dH_2_O) which was regarded as internal standard. After 30 s of vortex mixing, steel balls were added and grinded for 4 min at 45 Hz followed by treating with ultrasound for 5 min in ice water, then repeating this step for 3 times. The supernatant (0.5 mL) was transferred into a fresh 2 mL GC/MS glass vial after centrifuging for 15 min at 13,000 rpm, 4 °C. Next, the extracted metabolites were dried in a vacuum concentrator without heating and 30 μL of methoxy amination hydrochloride was added. After incubating in oven at 80 °C for 30 min, 40 μL of the BSTFA regent (1% TMCS, v/v) was mixed well with the sample aliquots and cultured for 1.5 h for 70 °C to obtain the derived metabolites for GC–MS analysis.

### GC-TOFMS analysis

GC-TOFMS analysis was performed using an Agilent 7890 gas chromatograph system coupled with a Pegasus HT time-of-flight mass spectrometer. The system utilized a DB-5MS capillary column coated with 5% diphenyl cross-linked with 95% dimethylpolysiloxane (30 m × 250 μm inner diameter, 0.25 μm film thickness; J&W Scientific, Folsom, CA, USA). A 1 μL aliquot of the analyte was injected in splitless mode. Helium was used as the carrier gas, the front inlet purge flow was 3 mL min^−1^, and the gas flow rate through the column was 1 mL min^−1^. The initial temperature was kept at 80 °C for 1 min, then raised to 290 °C at a rate of 10 °C min^−1^, then kept for 12 min at 290 °C. The injection, transfer line, and ion source temperatures were 280, 295, and 220 °C, respectively. The energy was − 70 eV in electron impact mode. The mass spectrometry data were acquired in full-scan mode with the *m/z* range of 50–600 at a rate of 12.02 spectra per second after a solvent delay of 8.45 min.

## Results

### Metabolic profiling of gas plasma-treated cells samples by GC-TOF

**Fig. 1:**
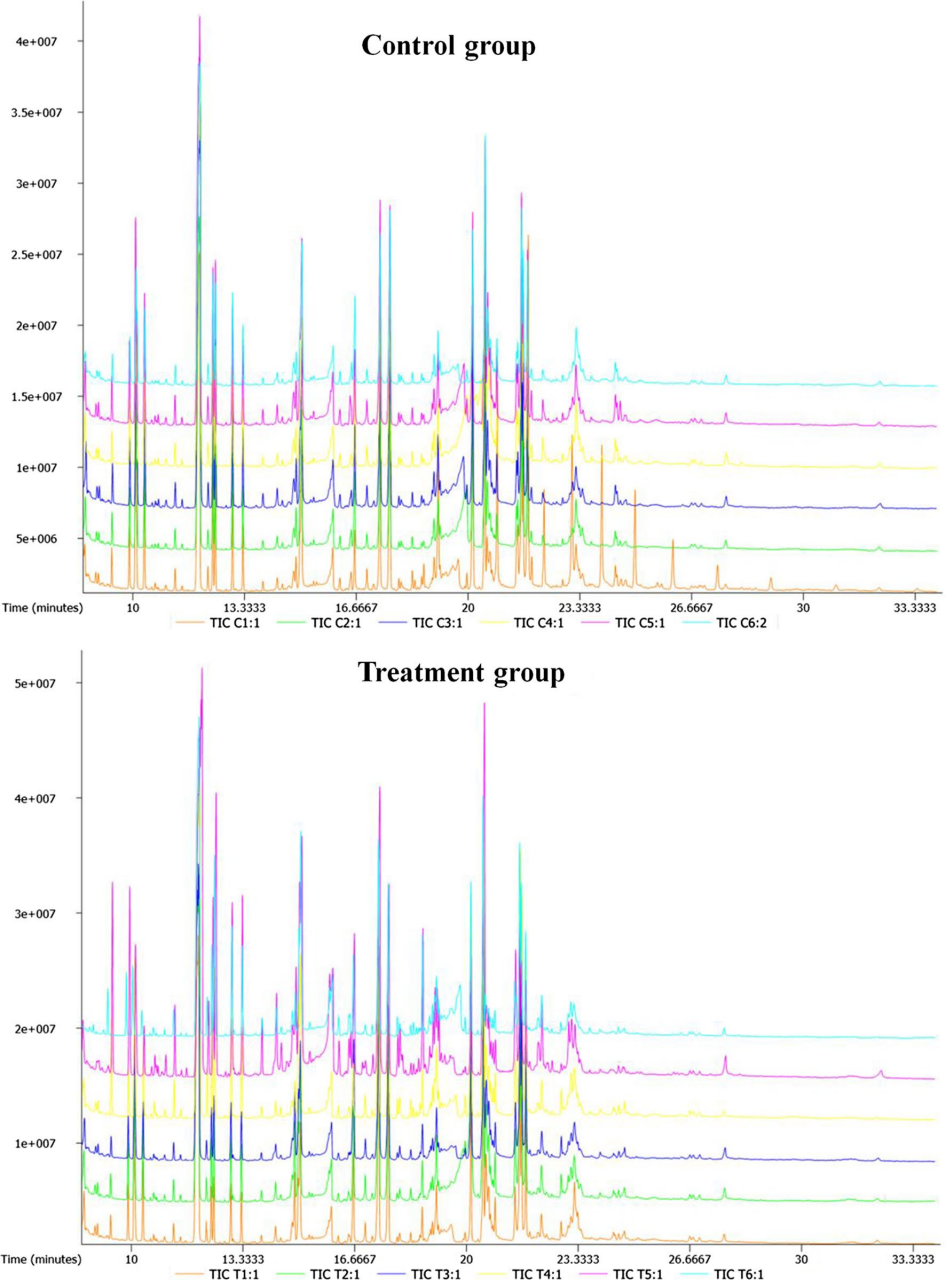
Representative GC-TOF chromatograms of control group and plasma treatment group by mass spectra

We investigated a total of 12 samples. Six were LP-1 cells with gas flow as control group, another six samples were LP-1 cells treated with He plasma for 1 min. With gas chromatography-time of flight mass spectrometry (GC-TOF), around 573 signals were detected per sample using mass spectral deconvolution software for peak detection. However, some of these signals were not consistently found in other samples or were of too low abundance or too poor spectral quality to be unambiguously assigned to unique metabolites. We have normalized with internal standard (IS) and finally 561 valid peaks were remained for further analysis. Details of these 561 peaks were listed in Additional file 1: Table S1. Figure 1 shows the overall representative GC-TOF chromatograms of control group and plasma treatment group.

### Unsupervised evaluation of metabolite signatures using PCA and OPLS-DA

After obtaining the raw data, we carry out a series of multivariate variable pattern recognition analysis, which is the principal component analysis (PCA). Using the SIMCA software (V14.1, MKS Data Analytics Solutions, Umea, Sweden), the data is logarithmic (LOG) formatted (CTR) formatted and then automatically modeled [6]. The parameters of the PCA model are shown in the statistical model parameter Table 1. Since the two groups of samples are not very significant, the sample is basically in the 95% confidence interval (Hotelling’s T-squared ellipse), this data need to be further analyzed.

**Table 1:** Statistical model parameters table of PCA model

Model	Type	A	N	R^2^X (cum)
Model 1	PCA	3	12	0.569

Therefore, we use the orthogonal least squares—discriminant analysis (orthogonal projection to latent structures-discriminant analysis, OPLS-DA) statistical methods to further analyze the results. Through the OPLS-DA analysis, we can filter out the orthogonal variables that are not related to the categorical variables in the metabolites, and analyze the differences between the non-orthogonal and orthogonal variables to obtain the more reliable metabolites. LOGG software was added to the data using SIMCA software (V14.1, MKS Data Analytics Solutions, Umea, Sweden). First, the first principal component was analyzed by OPLS-DA model. The quality of the model was verified by sevenfold cross validation (model-to-class variability Y) and Q2 (predictability of the model) to determine the validity of the model by cross validation. Finally, Permutation test), randomly different times to change the order of the variable Y order to get a different random Q2 value, the model validity for further testing. For more information on the OPLS-DA model, refer to the statistical model parameter Table 2.

**Table 2:** Statistical model parameters table of OPLS-DA model

Model	Type	A	N	R^2^X (cum)	R^2^Y (cum)	Q^2^ (cum)
Model 2	OPLS-DA	1 + 1 + 0	12	0.486	0.985	0.81

**Fig. 2:**
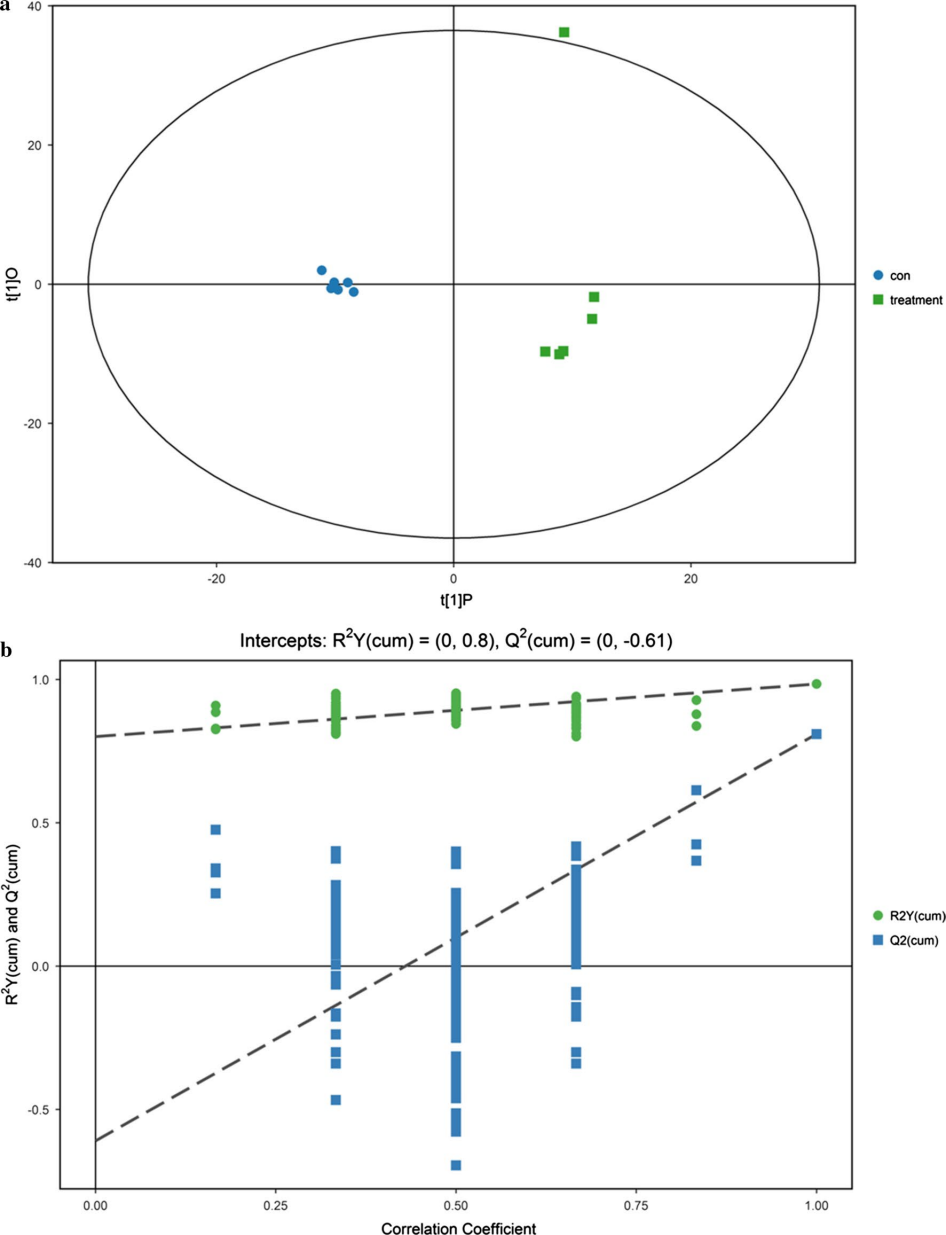
The score scatter plot of (**a**) OPLS-DA model and permutation test of (**b**) OPLS-DA model

As shown in Fig. 2a, the abscissa t [1] P represents the predicted principal component score of the first principal component, and the ordinate t [1] O represents the orthogonal principal component score, and the scatter shape and color represent different experimental groups. It shows that the two groups of samples are more significant, the sample is basically in the 95% confidence interval (Hotelling’s T-squared ellipse). The permutation test establishes the corresponding OPLS-DA model to obtain the random model R2 and Q2 values by randomly changing the permutation order of the categorical variable Y, the number of times (n = 200), the avoidance of the over-fitting of the test model and the evaluation of the model Significance has an important role. Figure 2b shows the replacement test for the OPLS-DA model.

### Identification of differential metabolites by supervised analysis

**Fig. 3:**
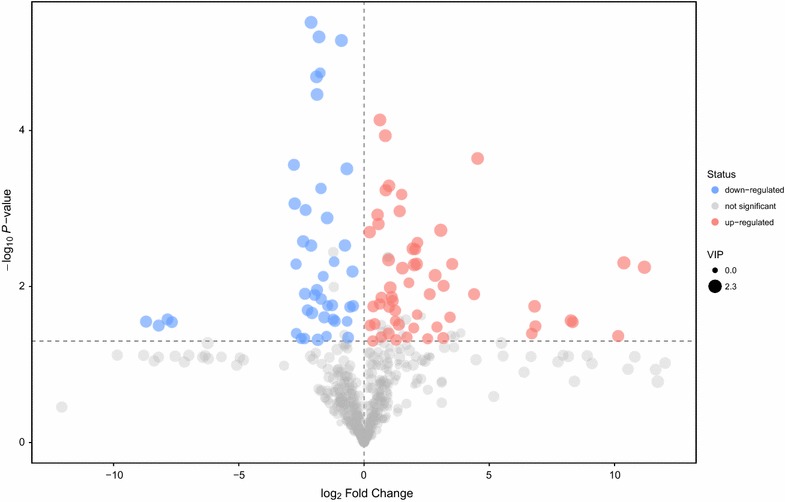
Volcano plot for differential metabolites between control and plasma treatment group

We used the generally recognized standard by academia, that is P value of student’s t test is less than 0.05, and the variable importance in the projection (VIP) of the first principal component of OPLS-DA model is greater than 1, to determine the differential metabolites between control and plasma treatment group. Additional file 1: Table S1 lists all the differences in metabolite screening, and was further illustrated in the form of volcano plot (Fig. 3). As shown in the final screening result, the significant uptake of the metabolites was displayed in red, while the significant down-regulation of the metabolites was shown in blue.

### KEGG analysis of differential metabolites

The complex metabolic responses and their regulation in organisms are not carried out separately, and often complex pathways and networks are formed by different genes and proteins. Their interaction and mutual regulation eventually lead to systemic changes in the metabolic group. The analysis of these metabolic and regulatory pathways can provide a more comprehensive and systematic understanding of the biological processes such as changes in the biological processes, the pathogenesis of the disease or the mechanism of the drug.

The kyoto encyclopedia of genes and genomes (KEGG) pathway database (http://www.kegg.jp/kegg/pathway.html) is based on the functional information of genes and genomes, and the metabolic response is clues, the possible metabolic pathways and the corresponding regulatory proteins, in a graphical way to show the cell physiological and biochemical processes. First, we mapped all the 561 metabolites to the Homo sapiens in the KEGG PATHWAY database. The mapping results are shown in Additional file 1.

**Fig. 4:**
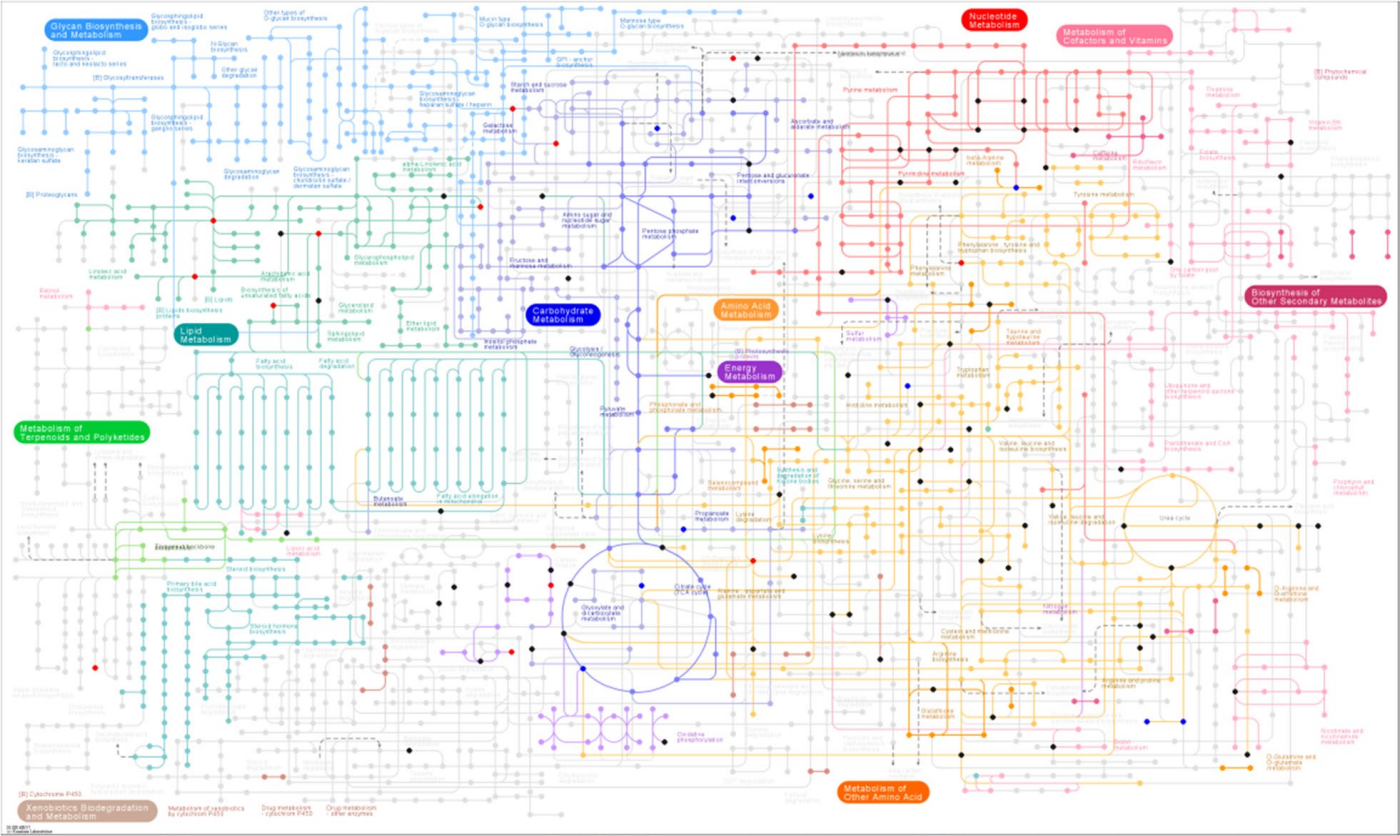
Metabolic pathways with red/blue dots representing the differentially expressed compounds

Based on the mapping results, we sort out all the pathways for the differential metabolite mapping, as shown in Additional file 2. After that, we labeled the differential metabolites on the KEGG pathway. As shown in Fig. 4, red represented up-regulation while bright blue represented down-regulation. And the black indicated that metabolites were detected but not significantly different.

### Metabolic pathway analysis of differential metabolites

**Fig. 5:**
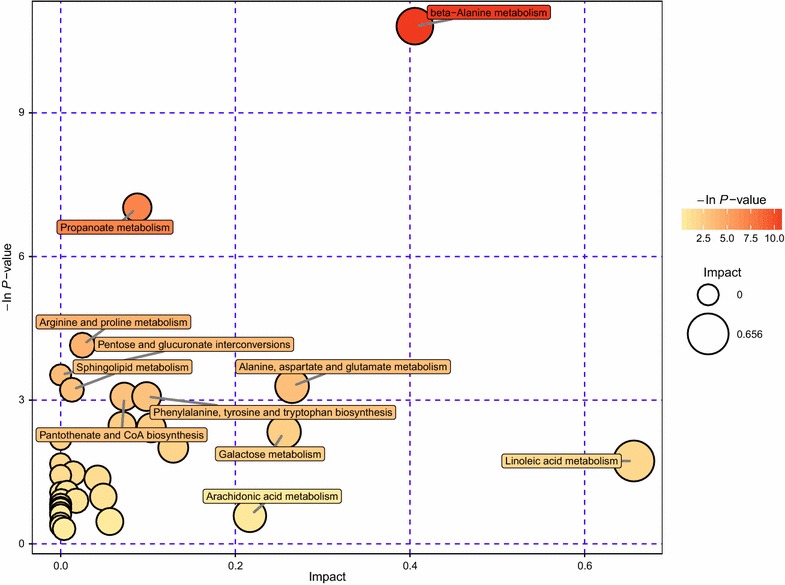
Bubble chart of the metabolic pathway analysis

KEGG analysis only found all the pathways that was relevant to these differential metabolites, to further analyze the most relevant pathways associated with these metabolite differences, a comprehensive analysis of the pathways of differential metabolites (including enrichment analysis and topological analysis) was carried out. We mapped these differential metabolites to the metabolites database such as KEGG, PubChem et al. and the metabolite mapping table was shown in Additional file 3. After obtaining the matching information of the different metabolites, we searched and analyzed the metabolic pathways in Homo sapiens database. An example of a metabolic pathway analysis table is given in Additional file 4. The results of the metabolic pathway analysis were presented as a bubble chart (Fig. 5). Each bubble in the bubble diagram represents a metabolic pathway. The abscissa and bubble size of the bubble indicate the size of the influence factor in the topology analysis. The larger the size is, the greater the influence factor is. The vertical and bubble color of the bubble indicate the enrichment analysis P value (negative natural logarithm, that is-ln P value), the deeper the color P value is smaller, the more significant degree of enrichment. From this chart we could clear figure out that beta-alanine metabolism pathway was the most significant changes after He gas plasma treatment in LP-1 cells. Besides, propanoate metabolism and linoleic acid metabolism should also be concerned during gas plasma treatment of cancer cells.

### Hierarchical clustering analysis of differential metabolites

The differential metabolites obtained by the above analysis are often biologically consistent and functional similarity/complementarity, or positive regulation/negative regulation by the same metabolic pathway, which will present similar or opposite expression characteristics between different experimental groups. The hierarchical clustering analysis of these characteristics will clear classify the metabolites with the same and different characteristics between the experimental groups.

**Fig. 6:**
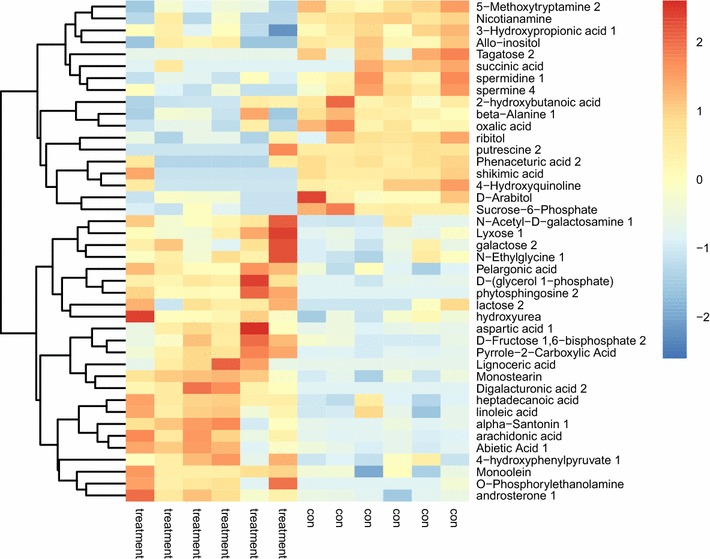
Heatmap of hierarchical clustering analysis for plasma treatment and control group

We calculated the Euclidean distance matrix from the plasma treatment group to quantify the differential metabolites of the control group. The results were visualized in a heatmap that was combined with hierarchical clustering of samples and metabolites (Fig. 6). The color patches at different positions represent the relative expression levels of the corresponding metabolites. It can be seen that there are obvious differences in metabolic grouping patterns after He plasma treatment.

## Discussion

Energy and material metabolism is the basic guarantee for cell survival. Adenosine triphosphate (ATP) is a currency in the cell that is used to store and deliver energy. In normal tissues, 90% of the ATP comes from oxidative phosphorylation, whereas only 10% comes from glycolysis [33]. In aerobic conditions, glycolysis is inhibited, known as the Pasteur effect. However, Warburg found that tumor cells are still prevalent with high rates of glucose uptake even under oxygenated conditions. The increased glycolytic metabolism and increased metabolites of lactate, which are ubiquitous in various tumor cells, called as the Warburg effect [34, 35]. Although the efficiency of glycolysis is low, tumor cells can benefit from glycolysis: Firstly, Due to the rapid growth of tumor cells, there is a great demand for energy and more glycolytic production of ATP is required. Secondary, glycolysis intermediates such as 6-phosphate glucose, pyruvate can synthesize fatty acids, nucleic acids which are important for cell metabolism and biosynthesis. Therefore, the energy and material metabolism of tumor cells and normal cells are quite different. Atmospheric cold plasma, as a newly developed technology, can selectively induce tumor cell death. In addition, some related apoptosis pathway factors were reported although more mechanism need to be investigated. In our study, instead of study on the single apoptotic protein, we investigated the whole metabolism profiling to understand the effect of plasma on the metabolism of tumor cells. Because the metabolomic data typically contains a large number of variables that are interrelated, multivariate statistical methods such as PCA and OPLS-DA were used in this study [36]. We demonstrated the large scale metabolic profiling using GC-TOF mass spectrometry and found numerous significant differences between the gas control group and the plasma treatment group in myeloma tumor cells. By KEGG analysis of the metabolic pathways we found that beta-alanine metabolism pathway was the most significant changes after He gas plasma treatment in myeloma LP-1 cells. Alanine, beta-alanine and sarcosine share the same chemical formula C_3_H_7_NO_2_, but are structurally different. By GC–TOFMS analysis, beta-alanine is easy to separate from alanine and sarcosine duo to its distinct mass spectrum [37]. Beta-alanine is a direct precursor of pantothenic acid (PA) which is needed for the synthesis of coenzyme A (CoA). In the tricarboxylic acid (TCA) cycle, CoA is important for pyruvate to enter as acetyl-CoA, and for α-ketoglutarate to be transformed to succinyl-CoA [38]. In addition, CoA is involved in the biosynthesis of many important compounds such as fatty acids, cholesterol, and acetylcholine [39]. Therefore, by He plasma treatment, beta-alanine metabolism in myeloma tumor cells was suppressed, which disturbing the energy and material metabolism of the tumor cells and results in tumor cells death. Our data illustrated some details about the dysregulation of metabolism profiling by gas plasma for the first time. Although more researches need to be done to further analyze the mechanism under molecular microstructure, this study gives a general direction for further study. Meanwhile, more tumor cell lines and the treatment by different types of gas plasma devices will be done for metabolite profiling analysis, to further illustrate the biological effects in various tumor cells by different reactive species in gas plasma.

## Conclusions

In conclusion, we demonstrated the effects of gas plasma on tumor cell metabolism by GC-TOF mass-spectrometry for the first time. By bioinformatics analysis we showed that plasma treatment could significantly alter the metabolite profiling of tumor cells. In addition, beta-alanine metabolism pathway was the most susceptible to plasma treatment, which might be instructive to further detail the mechanism of biological effects induced by plasma treatment in tumor cells.

## Additional files

**Additional file 1: Table S1.** KEGG metabolite mapping.

**Additional file 2: Table S2.** KEGG pathway.

**Additional file 3: Table S3.** Pathway metabolite mapping.

**Additional file 4: Table S4.** Pathway analysis.

## Abbreviations

MM: multiple myeloma
GC-TOF: gas-chromatography time-of-flight
CAP: cold atmospheric plasma
PCA: principal component analysis
VIP: variable importance in the projection
KEGG: kyoto encyclopedia of genes and genomes
ATP: adenosine triphosphate
PA: pantothenic acid
CoA: coenzyme A
TCA: tricarboxylic acid
